# Structural retrofitting of RC slabs using bamboo fibre laminate: Flexural performance and crack patterns

**DOI:** 10.1016/j.heliyon.2024.e23999

**Published:** 2024-01-04

**Authors:** Paul O. Awoyera, Fadi Althoey, Alireza Bahrami, Pius U. Apuye, L.M. Bendezu R, Badr S. Alotaibi, J.M. Prado M, Mohammed A. Abuhussain

**Affiliations:** a Department of Civil Engineering, Covenant University, Ota, Nigeria; b Department of Civil Engineering, Najran University, Najran, Saudi Arabia; c Department of Building Engineering, Energy Systems and Sustainability Science, Faculty of Engineering and Sustainable Development, University of Gävle, 801 76 Gävle, Sweden; d Department of Architecture, Ricardo Palma University, Lima, Peru; e Architecture Engineering Department, College of Engineering, Najran, Saudi Arabia

**Keywords:** Bamboo fibre laminate, Flexural performance, Crack pattern, Mode of failure, Sustainable material, Retrofit, RC slab

## Abstract

Enhancing the durability of structural elements is a viable approach to promote sustainability in civil engineering. Research has shown that well-maintained slabs outperform degraded ones, which deteriorate rapidly due to insufficient upkeep. The occurrence of cracking and deformation in slabs subjected to sustained loads significantly impacts their functionality. However, the implementation of appropriate retrofitting techniques utilizing locally available materials can effectively minimize deflection and crack propagation while also improving flexural capacity. This particular study aimed to evaluate the flexural performance of slabs that were retrofitted using bamboo fibre laminate (BFL). Also, the study investigated two alternative replacement methods alongside the conventional mix; one involved replacing all fine aggregates with ceramic fine aggregate and the other involved a complete replacement of coarse aggregates with ceramic coarse aggregate. These mixes were represented in both the retrofitted and non-retrofitted samples. The retrofitting process included using the combined external bonding and near surface-mounted method. Twelve slab samples were made, with six being non-retrofitted and the other six retrofitted with BFL. Each of the samples had dimensions of 300 mm × 300 mm × 50 mm for reinforced concrete (RC) slabs. The slabs were tested employing the three point-bending system, and the retrofitted slabs with the conventional mix exhibited the highest ultimate failure load and flexural strength (62.1 kN), which compared to the non-retrofitted slabs of the same mix was a 60.76% increase. Additionally, the study did a thorough analysis of the presence of flexural and diagonal shear cracks, as well as the occurrence of debonding between BFL and the slabs. Non-destructive tests were also conducted on the slab samples to further confirm accurate results. These findings offer helpful insights into the development and application of a sustainable retrofitting material that can remarkably improve RC slabs.

## 1 Introduction

Reinforced concrete (RC) is a core material used in constructing structural members. It is a homogeneous material, made up of concrete with steel reinforcement bars embedded in it. Concrete is high in its compressive strength but low in tensile strength. Steel reinforcements are high in tensile strength. Structures including foundations, beams, columns, and slabs are built using RC [1].

Slab failures in buildings, bridges, and other structures can occur for a variety of reasons. For instance, punching shear failure can occur in a conical shape, which causes a slab to dramatically collapse progressively after the induced load is redistributed, and consequent overloading the various structural elements within the system [2,3]. Also, several factors influence the flexural performance of RC slabs, including structural element size, cement, aggregate, moisture level, and ambient environmental conditions [[4], [5], [6]].

RC structures often need to have their performance improved over their useful lifetimes. When considering whether or not to retrofit a building, factors other than substandard materials and damaged structural components are considered. Changes of a building's function, environmental condition, or applicable building code could all be reasonable grounds to retrofit a building.

Retrofitting is the technique of enhancing the performance of structural components or existing structures with new technologies, features, and details. Retrofitting entails making repairs, rebuilding, or reinforcing an existing RC structure. Several techniques are used throughout the retrofitting process, including grouting, near-surface-mounted external post-tension, section expansion, fibre-reinforced polymer (FRP) composites, and external plate bonding.

There are two categories for retrofitting: local retrofitting and global retrofitting. The universal retrofitting approach is centred on the structures' seismic resistance. It also includes filling structure addition and foundation isolation. Shear members can be added to construction using flat slabs or flat plates. They can be provided in outside frames with the least degree of disruption to the building's functionality. The seismic resistance of a member is the objective of a local retrofitting procedure. Using concrete, steel, or FRP, the local retrofitting process includes structural elements such as beam-column joints, foundations, and column-beam connections. The process of laying a fresh layer of concrete with longitudinal reinforcement and evenly spaced connections is known as concrete cladding. The jacket improves the column or beam's flexural strength in both directions [7,8].

Researchers [7,[9], [10], [11]] have explored various synthetic fibres for structural remediation in buildings and obtained useful results. However, natural fibres are materials that are ecologically friendly and renewable, and have adequate structural qualities. Natural fibres are categorised into three groups depending on their sources: minerals, protein-containing animal parts like silk, hair, and wool, as well as plant fibres like hemp, flax, and bamboo. Cellulose, hemicellulose, lignin, and pectin are the primary substances found in plant fibres [[12], [13], [14], [15]].

Bamboo is the most significant plant fibre because it is environmentally beneficial, has a rapid growth rate, and absorbs carbon dioxide from the atmosphere. There are more than a thousand species of bamboo found around the world [16]. A few advantages of bamboo are its accessibility, affordability, safety for the environment while being used, and speedy pace of development. It takes bamboo less time to develop to its full capacity.

Bamboo can grow between 80 and 300 mm per day. Further enhancements to the bamboo's performance are possible when it is reshaped into a plate form. As a result, bamboo has a highly bright future in applications involving mass building [17].

Flexural strength plays a crucial role in the design and execution of various concrete structures, including slabs [18]. The significance of the flexural strength in slabs cannot be overstated when it comes to ensuring the structural integrity of buildings and infrastructures. It serves as a crucial factor in enabling these structures to withstand external forces, including various loads. Additionally, the flexural strength plays a pivotal role in preserving the integrity of structures by determining their capacity to resist deformation. Therefore, comprehending this aspect and exploring methods to enhance the flexural strength is essential for optimal functioning of buildings and ensuring the safety of occupants [19]. In this study, the focus includes determining the physical and mechanical properties of raw materials, examining the resistance of retrofitted and non-retrofitted slabs to flexural loading experimentally, and evaluating the failure pattern of the tested slabs. The results are expected to be very useful for engineers and contractors working on innovative solutions to building problems.

### 1.1 Retrofitting methods

The potential for the FRP attachments to come apart from the surrounding concrete surfaces is one of the key drawbacks of adopting FRP reinforcing systems. Since debonding is known to occur at low axial strain levels of FRP, externally bonded systems usually do not utilize the full tensile strength of FRP [20]. Recent years have seen an increase in research into and use of near-surface mounted (NSM) FRP reinforcement. To attach the FRP reinforcement to an RC element using the NSM technique, grooves must first be cut into the concrete cover of the element (often epoxy paste or cement grout). Groove filler serves as an interface for the distribution of stresses between the FRP bar and concrete. The FRP strip or rod is effectively fixed to concrete by adhesive in groove to serve as a tensile or shear reinforcing element. Cement paste or mortar has recently been assessed for its impact, capacity to increase bonding to wet substrates, and ability to withstand high temperatures to replace epoxy as a groove filler and minimize material costs and environmental impact [21]. NSM has demonstrated to make greater use of bonded FRP materials than the other strengthening methods [22]. The NSM approach is a cutting-edge technique that offers a high level of strengthening performance, is less likely to experience early debonding failure, and increases resistance to fire, mechanical damage, effects of ageing, and vandalism-related activities. Given that, the reinforcement is internal, the approach also provides better resilience, stress-sharing mechanisms, and fatigue performance [23].

Having these constraints, a hybrid strengthening strategy combining the external bonding reinforcement (EBR) and NSM approaches is proposed. The strengthening approach also referred to as the combined externally bonded and near-surface mounted (CEBNSM) strategy, offering a clever and optimal combination of the EBR and NSM procedures that function to complement one another and do away with each other's limitations [24].

### 1.2 Previous studies

Several studies have been conducted on the use of FPRs in structural applications for the flexural performance.

Nambiyanna et al. [25] studied the flexural strengthening of RC slabs using Jute and Coir as natural fibre composites. It was observed that the load-carrying capacity of reinforced slabs increased compared to regular slabs. Cracking load increased by 6.67–33.33%, and ultimate load enhanced by 15.03–37.25%. Moreover, it was witnessed that Jute and Coir fibre-reinforced slabs had better post-performance than regular slabs.

The flexural performance of externally bonded bamboo laminates on prestressed concrete hollow-core slabs was investigated by Wang et al. [26]. It was concluded that the application of externally bonded laminated bamboo plates effectively strengthened precast hollow-core slabs. The use of laminated bamboo, which has lower stiffness, helps prevent debonding issues and allows the full strength of the laminate to be utilized.

Fang et al. [27] carried out a study on composite fibre-reinforced grid facesheet slabs, utilizing a glass FRP (GFRP) grid as a reinforcement system. It was revealed that narrowing the rib spacing of the GFRP grid led to substantial enhancements in the bending stiffness and ultimate load-carrying capacity of the composite slabs. These findings offer valuable insights into the design and optimization of composite slab systems, contributing to the advancement of structural engineering practices.

Lv et al. [28] reported that the ultimate load-carrying capacity of cross-laminated bamboo slabs without carbon FRP (CFRP) grids exhibited a positive correlation with layer thickness and the number of layers. Furthermore, the introduction of CFRP grids through pressing into the bamboo layer substantially increased the ultimate load-carrying capacity. However, the presence of CFRP grids bonded in the interface of adjacent layers negatively impacted the load-carrying capacity, suggesting the need for careful consideration during construction.

Lim et al. [29] evaluated the influence of steel sheet profile and thickness on the flexural behaviour of composite slabs. The findings indicated that composite metal decks outperformed conventional slabs in terms of the flexural performance, incorporating a steel sheet profile which considerably increased the ultimate load-carrying capacity (537.4%) and ductility (100.9%) of the conventional slab, thereby pointing out the potential of composite structures in reducing the reliance on concrete and steel reinforcement.

To examine the flexural behaviour of an RC beam strengthened with Kenaf FRP laminate, a thorough experimental and numerical analysis was done by Nwankwo and Ede [30]. Adding the KFRP laminate greatly boosted the RC beam's ability to carry loads by 77.9%. In contrast to the control beam, the KFRP-strengthened beam was more brittle since this increase in the strength was at the expense of the decreased ductility.

Aman et al. [31] illustrated that adding more CFRP layers to RC slabs enhanced their load-carrying capacity and decreased their deflection. It was displayed that the load-carrying capacity of the slabs improved within a range of 4%–9% with each additional CFRP layer. However, it was observed that the load-carrying capacity decreased by up to 7% as the size of the opening increased to its maximum.

Near-surface mounted carbon fibre reinforced polymer (NSM-CFRP) has been studied as a potential reinforcement material for prestressed hollow-core slabs [32]. They came to the conclusion from their trials that NSM-CFRP strengthening significantly increased prestressed hollow-core slabs' flexural and shear capacities. When compared to the unstrengthen slabs, the reinforced slabs showed an increase in cracking load of 4–9%. However, in slabs that collapsed owing to shear before attaining their maximum flexural capacity, the level of improvement in the ultimate load-carrying capacity decreased.

## 2 Materials and methods

### 2.1 Materials

**Fig. 1:**
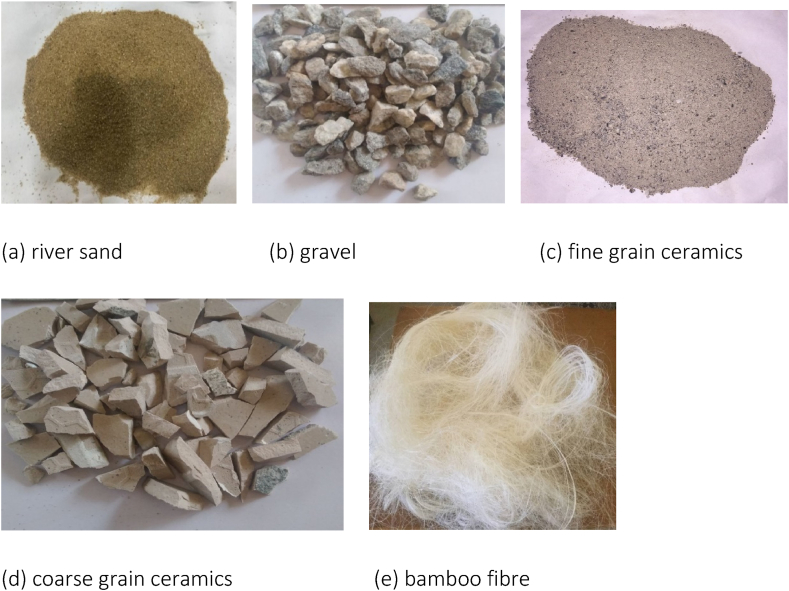
Materials for fabrication of slabs.

The materials utilized in this study included grade 42.5 ordinary Portland cement, which falls in the category of an ASTM C150 standard type I multipurpose cement. Other materials were river sand as fine aggregate (< 4.75 mm), gravel as coarse aggregate (8–12 mm), and ceramics. Water for mixing was pure, colourless, freshwater, and odourless in accordance with specifications from ASTM C1602. Bamboo fibre was used for laminate fabrication. Fig. 1 depicts the materials used for fabrication of slabs.

The reinforcement provided for the slabs was 5 bars of 6 mm diameter, spaced at 50 mm, spanning in both directions of the slabs. The bonding behaviour between bamboo FRP and the concrete substrate was improved by the application of epoxy resin. Because of the superior corrosion and solvent resistance, tolerance to high temperatures and heat, strong fatigue resistance, and high modulus of elasticity of epoxy resin, it offers a good bond between the concrete substrate and bamboo FRP. Sikadur was also used, which is a two-component adhesive and repair mortar based on epoxy resins and unique fillers. It is employed to fill gaps, cavities, joints, and fractures on vertical and overhanging surfaces, and it is also a structural adhesive and mortar for concrete elements, hard natural stone, ceramics, and fibre cement. The mechanical properties of Sikadur, obtained from the manufacturer, are presented in Table 1.

**Table 1:** Mechanical properties of Sikadur 31 CF cured at 23 °C.

Property	Value
**Tensile strength**	∼ 18–24 N/mm^2^
**Compressive strength**	∼ 60–70 N/mm^2^
**Modulus of elasticity in compression**	∼ 4600 N/mm^2^
**Modulus of elasticity in tension**	∼ 5000 N/mm^2^
**Flexural strength**	∼ 30–40 N/mm^2^
**Tensile strength of adhesion (concrete dry)**	∼ 2 N/mm^2^
**Pot life**	∼ 55 min
**Curing time**	7 days
**Mixing ratio**	Component A:Component B = 2:1 by weight or volume

### 2.2 Methods

#### 2.2.1 Batching of concrete constituents

In this research, batching of materials was done by weight. M20 1:1.5:3:0.5 mix ratio (cement: fine aggregate: coarse aggregate: water) was adopted because the standard mix ratio for slabs is between M20 and M25. For casting, a timber formwork mould was used, and the mould was adequately lubricated for the easy removal of hardened concrete.

The concrete constituents were according to the mix ratio and 300 mm × 300 mm × 50 mm specifications. Thus, for a typical slab, the utilised constituents were cement content (kg): 4.5, fine aggregate (kg): 6.75, coarse aggregate (kg): 13.5, and water (kg): 2.25.

Table 2 lists the cast slabs in this research. Each slab had an identical sample made to achieve the results more accurate.

**Table 2:** Slabs for experiment.

Designation of slabs	Description	Used mix
**NS1A & NS1B**	Control slab	Cement: Sharp sand: Gravel
**NS2A & NS2B**	Non-retrofitted slab with ceramic concrete fine aggregate replacement	Cement: Fine ceramic concrete: Gravel
**NS3A & NS3B**	Non-retrofitted slab with ceramic concrete fine aggregate and coarse aggregate replacement	Cement: Fine ceramic concrete: Coarse ceramic concrete
**RS1A & RS1**B	Retrofitted slab with conventional mix	Cement: Sharp sand: Gravel
**RS2A & RS2B**	Retrofitted slab with ceramic concrete fine aggregate replacement	Cement: Fine ceramic concrete: Gravel
**RS3A & RS3B**	Retrofitted slab with ceramic concrete fine aggregate and coarse aggregate replacement	Cement: Fine ceramic concrete: Coarse ceramic concrete

Each slab sample was cast in pairs for better use in evaluating the results of the experiments. The non-retrofitted slab consisted of six samples and the retrofitted slab also included six samples. In the designations of the slabs, 1A and 1B indicate the presence of nominal conventional mix used to cast slabs, while 2A and 2B illustrate the use of ceramic concrete fine aggregate as a replacement for sharp sand. Finally, 3A and 3B utilised ceramic concrete fine aggregate and coarse aggregate as a 100% replacement for sharp sand and gravel, respectively.

#### 2.2.2 Sieve analysis

The grade of the materials that was employed as aggregates was determined by this test. It ensures that the distribution of particle sizes conforms with the relevant standards and provides the details required to manage the material of different aggregate products and combinations incorporating aggregates. The apparatus used were standard sieves, mechanical sieve shaker, weight balance, electric oven, and sample splitter.

#### 2.2.3 Slump test

This test was used to measure the workability of freshly mixed Portland cement concrete in the lab. It determines the consistency of concrete as well as how readily it can be placed or vibrated.

#### 2.2.4 Casting of slab

**Fig. 2:**
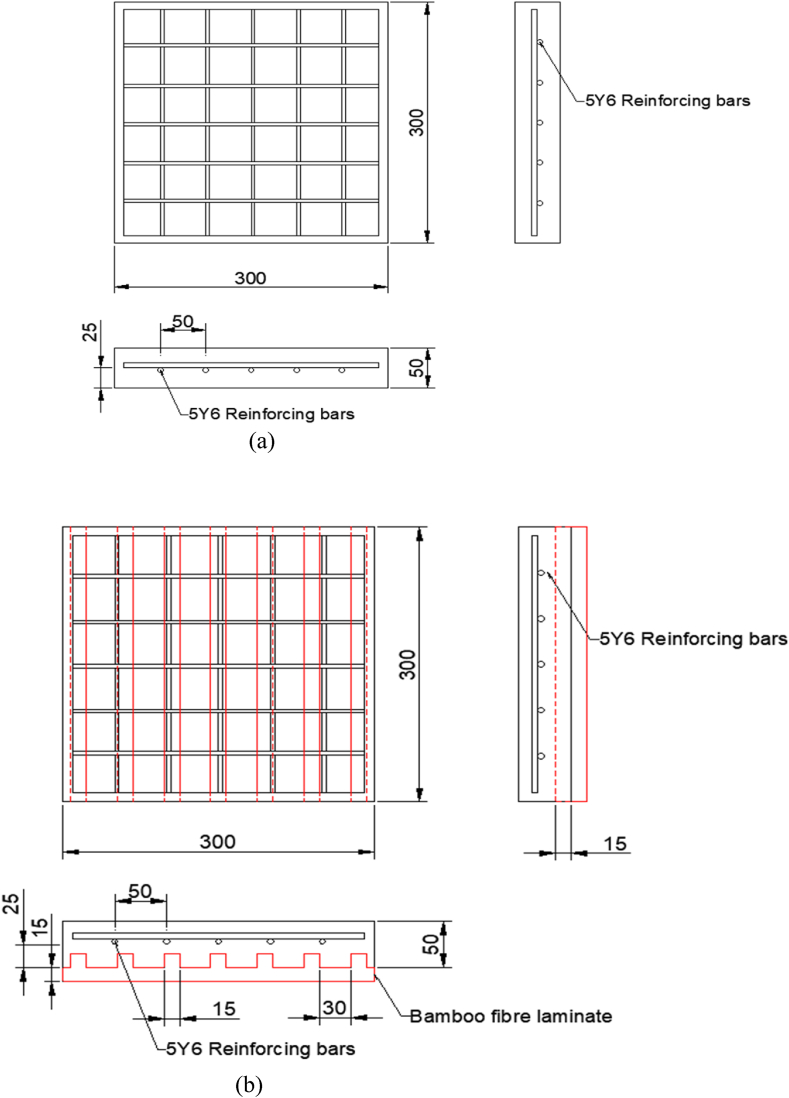

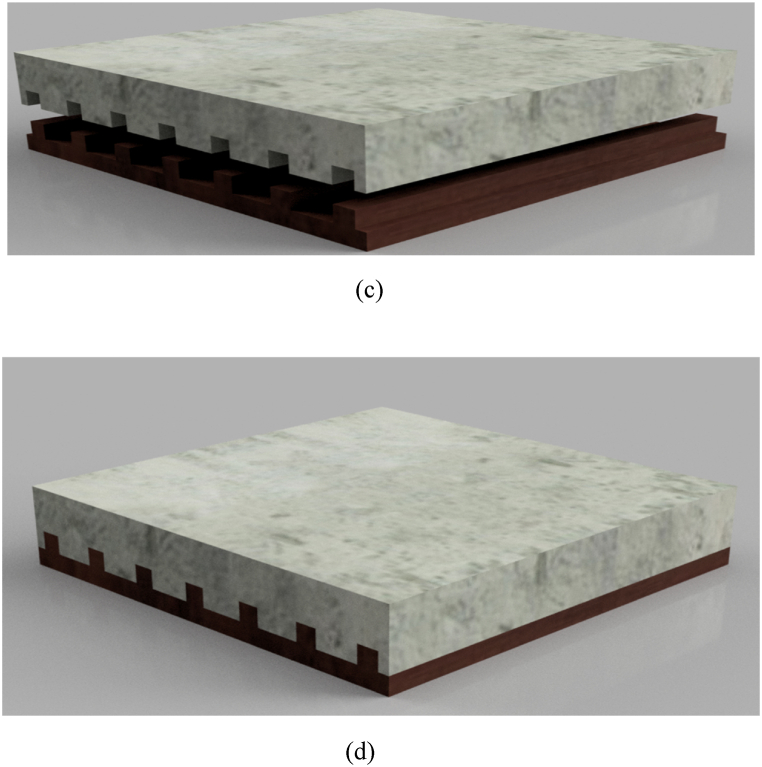
(a) Plan, front view, and side view of slab and reinforcements (dimension: mm), (b) Plan, front view, and side view of slab, bamboo fibre laminate (BFL) retrofit, and reinforcements (dimension: mm), (c) 3D view of BFL and slab, (d) 3D view of joined BFL and slab.

This research required the use of 12 slab samples. The mixing of concrete was done manually. The constituents were mixed until a homogenous substance was formed. Three 50-mm layers of concrete were poured, each of which was tamped 25 times with a 12-mm tamping rod. In the formwork, there were 5 bars of 6 mm in diameter, with a spacing of 67.5 mm placed in both directions and soldered to hold reinforcements in place. Concrete biscuits of 15 mm in size were added to ensure the reinforcing bars did not touch the formwork and also ensured a 25 mm concrete cover in all directions of the slabs. Concrete was then poured into a rectangular formwork with the required specifications and allowed to set. After 24 h, the slabs were de-moulded and placed in a curing tank for 28 days to ensure their maximum strength was reached. The schematic views of the slab and reinforcements are displayed in Fig. 2.

### 2.3 Retrofitting methods

#### 2.3.1 EBR method

To facilitate the easy removal of BFL, a mould was prepared based on the slab specifications and adequately lubricated with oil. To prevent the laminate from sticking to the mould, a nylon sheet was placed on top. The epoxy resin and hardener were mixed in a ratio of 2:1, and this mixture was applied to the mould. An appropriate amount of resin was added before and after each layer of bamboo fibres to ensure thorough saturation and eliminate any air pockets within the laminate. Once the desired amount of bamboo fibre was added, the mould was covered, and a load was applied on top. The mould was then left undisturbed for 48 h. The compression of the load aided in the curing process and promoted the better formation of the laminate mould, as the epoxy resin operates through heat transfer mechanisms. After the curing process was complete, the laminate was affixed to the slab using Sikadur 31-CF adhesive and compressed with an applied load. The slab sample was then allowed to cure for 7 days, optimizing the adhesive properties between the laminate and slab.

#### 2.3.2 NSM method

The following processes were carried out for the NSM method.

i. Placing of the groove mould: This step involved placing a square box of 15 × 15 mm in size. This was to give the groove shape needed for BFL before pouring concrete.
ii. Adhesive application: The base and hardener were combined to create epoxy, which was then applied to groove.
iii. Installation of the FRP strips: The BFRP strips were divided into the necessary strips, after which lengths and widths were put into the epoxy-filled groove.
iv. Applying adhesive to the top of the strips: The remaining strips were then thoroughly covered with another coat of epoxy.
v. Removing extra glue from surfaces: The extra adhesive was cleaned off to create the completed product from the groove sides.
vi. Curing: The retrofitted samples were then allowed to cure in the air.

**Fig. 3:**
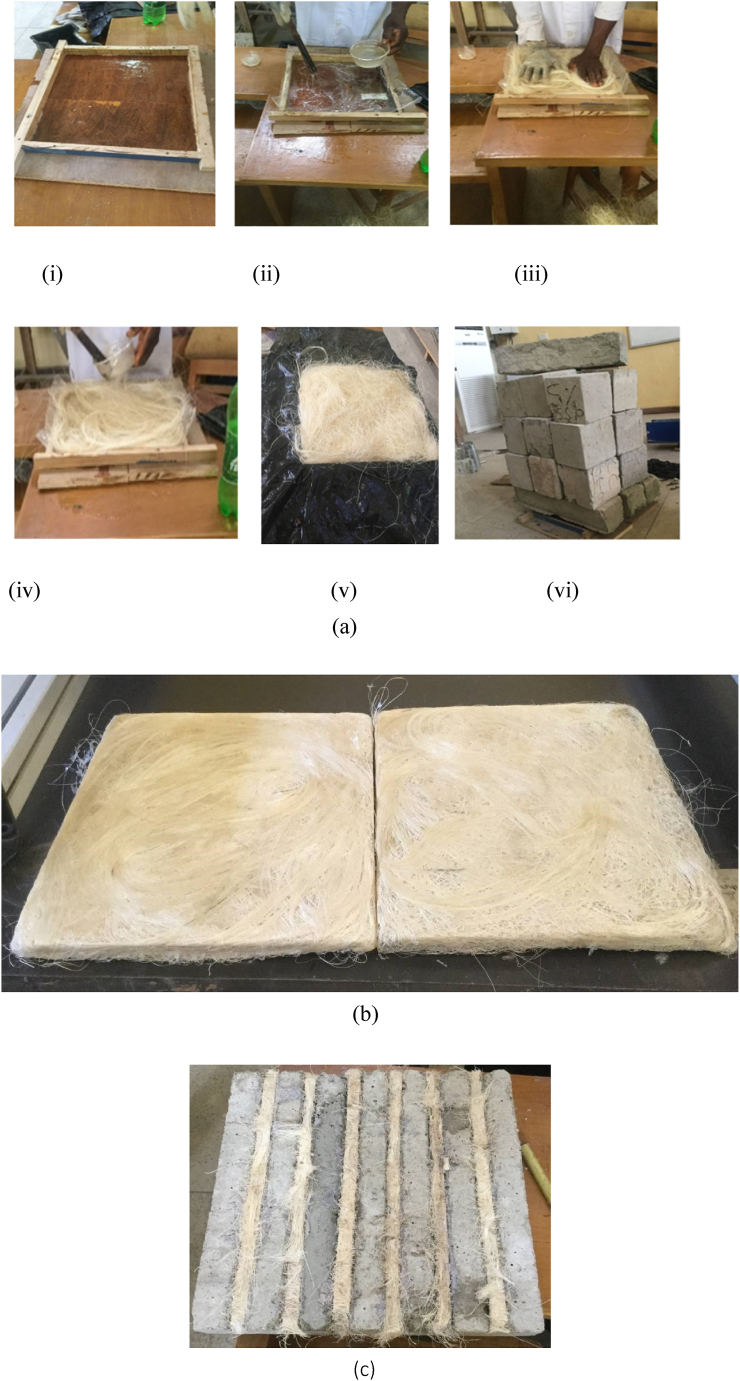
(a) Bamboo fibre formation process: (i) Lubricating formwork, (ii) Addition of epoxy resin and hardener (2:1), (iii) Addition of bamboo fibre to formwork, (iv) Addition of extra layer of resin and hardener, (v) Bamboo fibre before being wrapped with nylon, (vi) Application of weight to compress bamboo fibre. (b) Fabricated BFL. (c) BFL used in NSM for slab.

Fig. 3a and b demonstrate the step-by-step procedures taken to fabricate the laminates used in this study, and Fig. 3c depicts BFL used in NSM for the slabs.

### 2.4 Test on hardened concrete

#### 2.4.1 Compressive strength

**Fig. 4:**
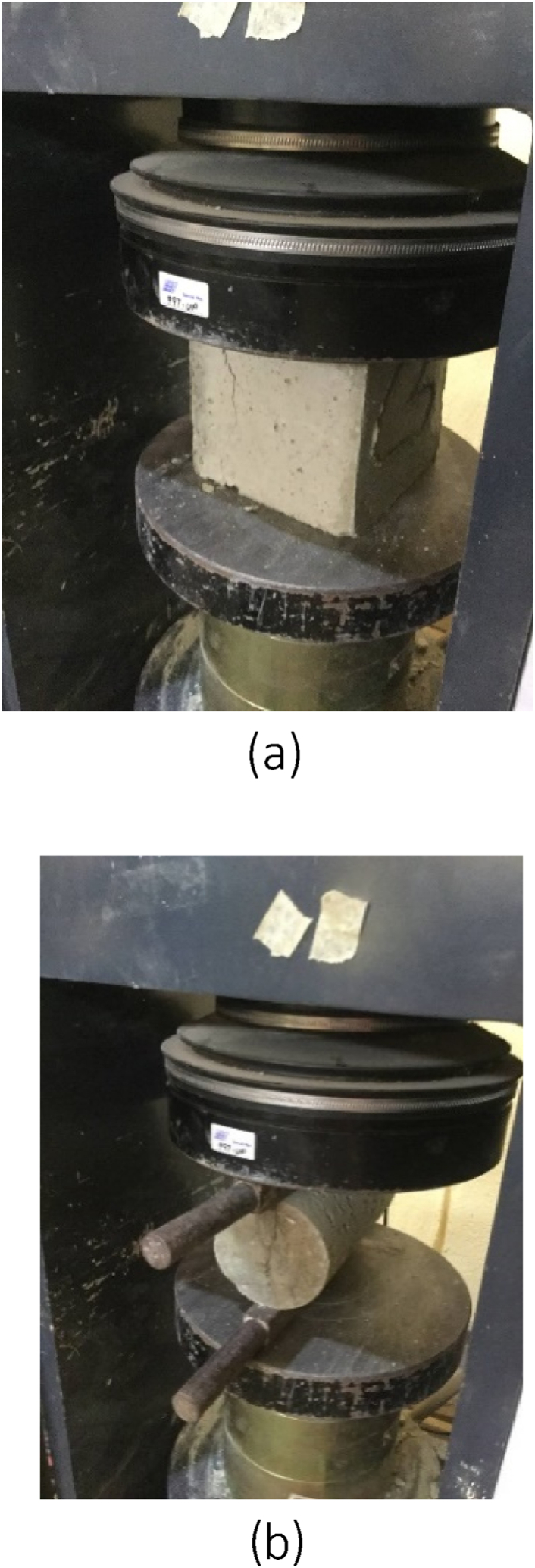
(a) Compressive strength test on cube sample, (b) Splitting tensile strength test on cylinder sample.

The ability of concrete to withstand a compressive load without failure is known as its compressive strength. To minimize the amount of voids, the test was conducted by appropriately compacting concrete after it was poured into a mould with the dimensions of 150 mm × 150 mm × 150 mm. The mould was removed 24 h later. The apparatus used was the compression testing machine (Fig. 4a).

#### 2.4.2 Splitting tensile strength

Splitting tensile strength is a property of concrete that measures its resistance to the tensile stress. It is defined as the maximum tensile stress that a cylindrical concrete sample can withstand before it splits apart along the vertical diameter. The splitting tensile strength of concrete affects the extent and size of cracking in structures. The apparatus utilised was the compression testing machine but with a different arrangement to accommodate cylindrical samples (Fig. 4b).

#### 2.4.3 Ultrasonic pulse velocity test

**Fig. 5:**
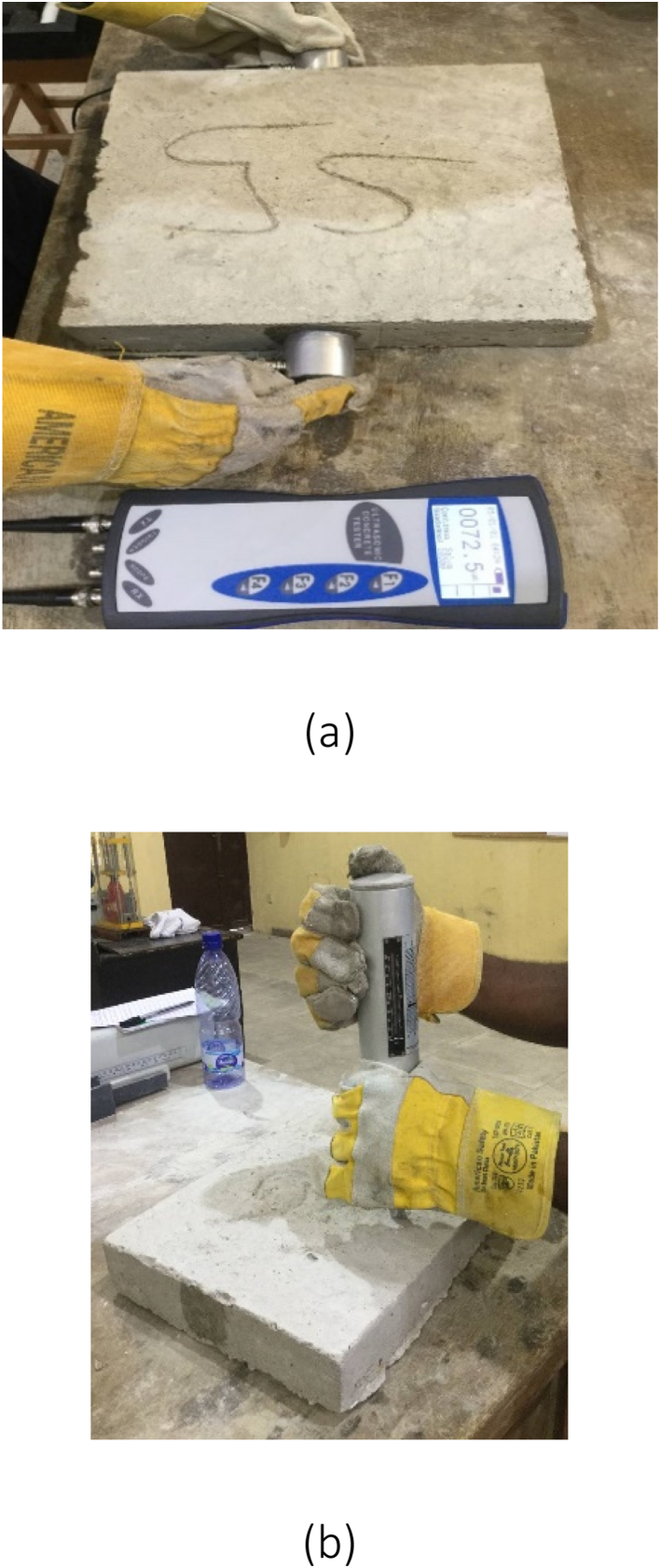
(a) UPV test on slab sample, (b) Use of rebound hammer on slab sample.

Ultrasonic pulse velocity (UPV) test is a non-destructive test used to check the quality, homogeneity, cracks, cavities, and defects in concrete. By monitoring the speed of an ultrasonic pulse as it passes through a concrete structure, this test determines the strength and quality of concrete. The test can be employed to determine the consistency and quality of concrete components, predict the concrete's strength, assess the concrete's dynamic modulus of elasticity, and obtain the extent of cracks in concrete. The apparatus utilised was the UPV tester. The test entails cleaning and drying the concrete surface to be tested and applying lubricant on the surface to ensure adequate smoothness followed by the placement of two transducers on the surface of concrete at a fixed distance apart (Fig. 5a). Practically, one transducer sends an ultrasonic pulse through concrete to the other transducer, and the time taken for the pulse to travel through concrete is measured, also known as the transit time. The velocity of the pulse is calculated using the distance between the transducers and the time taken for the pulse to travel between them.

The UPV test was conducted on the slabs without retrofitting using the direct transmission method. To initiate the ultrasonic waves, a pulse generator was employed. The slab sample allowed these waves to pass through. The ultrasonic pulses were transmitted and received by two transducers that were placed on the sample's opposing sides. The amount of the time needed for the pulse to pass through the slabs was measured, allowing for the computation of the pulse velocity using the following equation:

(1) *V* = *D*/*t*

This equation uses *V* to represent the pulse velocity, *D* to indicate the distance between the transducers, and *t* to designate the time.

To ensure the accuracy, the test was repeated several times, and a calculation of the concrete sample's average pulse velocity was made.

#### 2.4.4 Schmidt rebound hammer testing

It is a non-destructive test used to determine the compressive strength of concrete. During the test, the material's surface was struck by a spring-loaded hammer, and the hammer's rebound velocity was then calculated. The compressive strength can be determined by calculating the rebound velocity and comparing the result to a conversion table; this depends on the hardness of the material. The Schmidt rebound hammer testing equipment was used. The concrete's surface was cleaned, smoothed, and checked to make sure there was no loose material present before completing the test. The Schmidt hammer was calibrated utilising a calibration test anvil before the test was run. The calibration guarantees that the measurements from the Schmidt hammer are precise. The test's concrete surface was positioned perpendicular to the Schmidt hammer (Fig. 5b). The hammer should be set up so that the test area of the concrete surface will receive the majority of the impact. Each sample that underwent testing had its test results documented. Using a conversion table or equation, the rebound distance acquired from the Schmidt hammer was translated to a compressive strength value. In general, the values for the compressive strength might give a clue as to the strength and calibre of concrete. Higher rebound values signify higher strengths, whereas lower rebound values point out lower strengths.

#### 2.4.5 Flexural strength test

A common mechanical test for assessing the bending properties and strength of RC slabs and other structural elements is the flexural strength test. It is crucial for determining the slabs' structural integrity and load-carrying capacity in bending situations. The test entails applying a bending load to a slab sample until failure occurs, at which point the maximum load that the slab can withstand is recorded.

The results of the flexural strength test can be influenced by the slab sample's size and form, the quality and consistency of the concrete mix, and the testing circumstances such as the rate of loading and environmental elements. The American Society for Testing and Materials (ASTM) publishes standards such as ASTM C78/C78M − 18, the standard test method for the flexural strength of concrete, to ensure uniform and trustworthy testing processes.

**Fig. 6:**
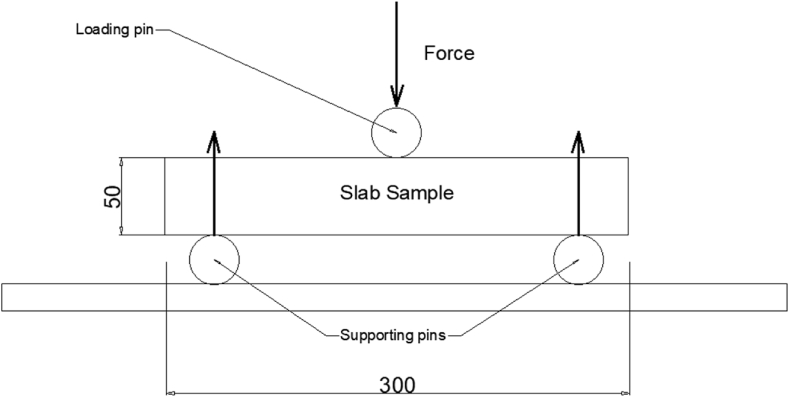
Technical setup of three-point bending system of flexural strength test on slab samples (dimension: mm).

To create test samples for the flexural strength test of the slabs, RC slabs were cut to the required size and shape mentioned in the testing standard. After that, the slabs were given time, 28 days, to undergo water curing. The samples were placed horizontally on the testing machine's supports with the load point in the middle of the samples following the curing period. The three-point bending test was used for this experiment and its technical setup is shown in Fig. 6.

The testing apparatus was adjusted so that the load was applied to the centre of the slab sample, and the space between the supports was adjusted in accordance with the sample's size and the specifications of the testing standard.

The slab sample was subjected to a continuous load during the test, which typically increased incrementally until the slab reached its maximum load-carrying capacity or failed. The greatest load that the slab sample could withstand was noted.

For the slabs to be able to carry their intended loads and achieve the necessary strength requirements, they must pass a flexural strength test. Accurate and trustworthy findings can be acquired for assessing the flexural strength of RC slabs by adhering to the standardized testing techniques established by ASTM or other pertinent authorities. The flexural strength of the slabs was obtained using equation (2).

(2) Flexural strength = (3*PL*)/(2*bd*^2^)

*P* = maximum load,
*L* = span between supports,
*b* = width of sample,
*d* = depth of sample.

## 3 Results and discussion

An explanation of the experimental data is presented here, along with the analysis and discussion of the obtained results. Through a variety of experiments, the emphasis was on analysing the mechanical and fresh qualities of concrete. A definitive conclusion was achieved after carefully analysing and thoroughly discussing the test data.

### 3.1 Sieve analysis

**Fig. 7:**
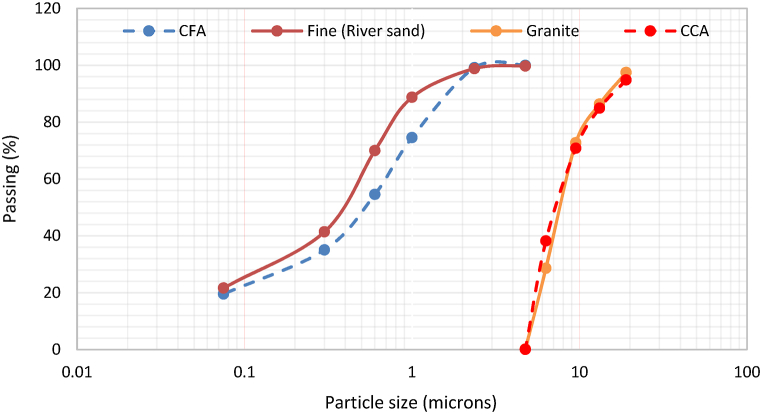
Particle size distribution of CCA, CFA, river sand, and granite.

In Fig. 7, the particle size distribution of different aggregates is displayed. Ceramic coarse aggregate (CCA), coarse natural aggregate (granite), ceramic fine aggregate (CFA), and fine aggregate (sand) were examined. Sand and CFA were sieved using a range of no. 4 to 100 sieves, with openings ranging from 2.36 mm to 0.075 mm. Granite and CCA were also sieved but with a larger openings of 19–2.36 mm. According to British Standards (BS), both granite and CCA are considered coarse. Similarly, sand used in the study is also classified as coarse based on BS.

The size gradient can be utilised in the determination of the grade of aggregate. Aggregate could be graded as either well-graded meaning the gradation is close to the maximum density gradation line, gap-graded meaning it has only a small percentage of particles in the mid-size range, uniformly-graded meaning it is composed of mostly particles of the same size or open-graded meaning it contains only a small percentage of small-sized particles. The particle size distribution curve of fine aggregate and CFA represents well-graded aggregate, hence, the gradation and distribution line are close to the maximum density gradation line. The particle size distribution curve of coarse aggregate and CCA indicates uniformly-graded aggregate, accordingly, the gradation is mainly composed of particles of the same size. The well-graded fine aggregate employed in conjunction with uniformly-graded coarse aggregate yields a highly workable concrete mix with minimal void spaces, consequently, making the grade of used aggregate adequately efficient for concrete.

### 3.2 Slump test

In this study, the focus was to assess the workability of freshly mixed concrete and investigate variations in the mix uniformity. To achieve this, we utilized the slump test method. Three different types of concrete mixes were employed: the control mix, a mix with 100% CFA replacement, and a mix using both CCA and CFA as substitutes for conventional aggregates.

**Fig. 8:**
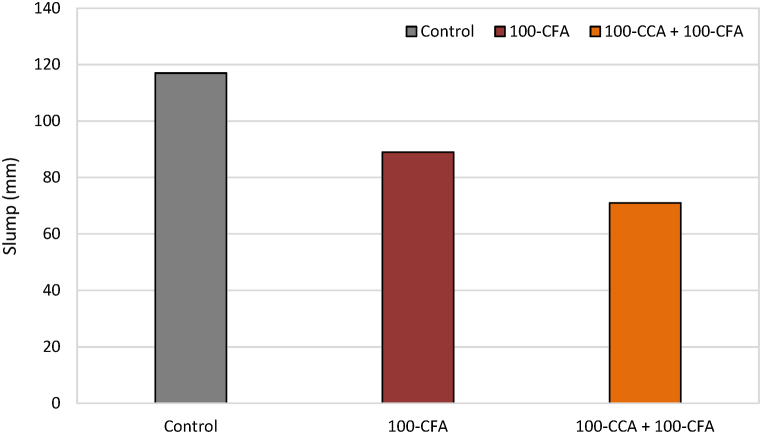
Results of slump test.

The results of the slump test, representing the workability of the fresh concrete mixes, are demonstrated in Fig. 8. It was observed that the control concrete, which had a water-to-cement ratio of 0.5, exhibited the highest slump value of 117 mm. The mix with 100% CFA replacement gave a slightly lower slump value of 89 mm. The lowest slump value of 71 mm was recorded for the mix with 100% CCA and CFA substitution. From the figure, it can be deduced that an increase in the percentage substitution of ceramic aggregates led to a reduction in the slump value.

The findings from the slump test pointed out that as the proportion of ceramic aggregates increased in the concrete mix, the workability decreased. Since the slump value serves as an indicator of the concrete's ability to flow and compact easily, therefore, the decrease in the slump values suggests a decrease in the workability with higher proportions of ceramic aggregates.

### 3.3 Test on hardened concrete

#### 3.3.1 Compressive strength

**Fig. 9:**
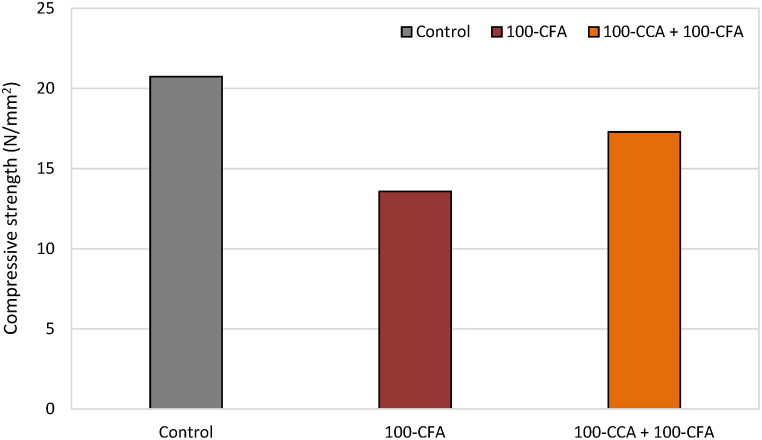
Average compressive strength results of concrete samples after 28 days of curing (N/mm^2^).

Fig. 9 depicts the compressive strength results of all the concrete mixes after a curing period of 28 days, using a water-to-cement ratio of 0.5. The compressive strength tests were conducted in accordance with the guidelines outlined in BS 1881: part 116. Concrete cubes measuring 150 × 150 × 150 mm were cast and subjected to testing to determine their strength at the end of the 28-day curing period. The testing was performed using a hydraulic universal machine with a capacity of 2000 kN and at a loading rate of 50 N in the load control mode. This equipment allowed the accurate measurement of the compressive strength of the concrete samples.

In accordance with Fig. 9, the highest average compressive strength was obtained by the control mix as 20.74 N/mm^2^. However, the lowest value was from the 100-CFA mix as 13.57 N/mm^2^.

#### 3.3.2 Splitting tensile test

**Fig. 10:**
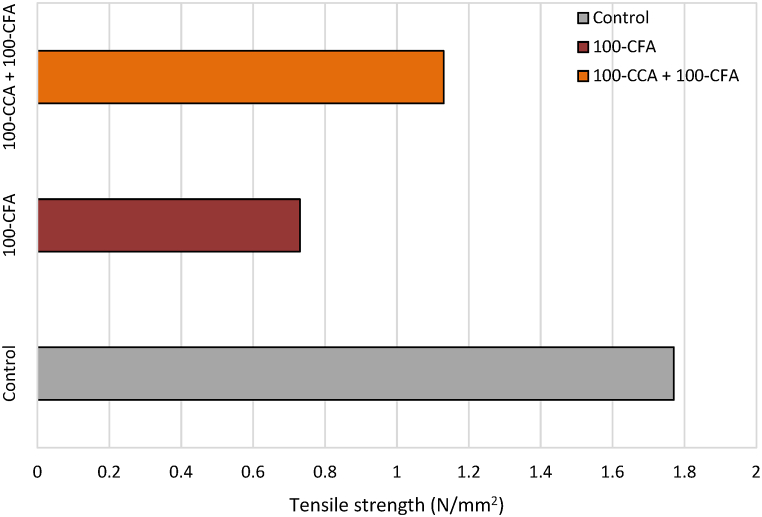
Average splitting tensile strength results of concrete samples after 28 days of curing.

A splitting tensile test was done on cylindrical concrete samples to assess their resistance to the tension. The samples consisted of a control mix, 100-CFA, and 100-CCA + 100-CFA. The splitting tensile strength results are illustrated in Fig. 10.

As can be seen in Fig. 10, the highest average splitting tensile strength was achieved by the control mix as 1.77 N/mm^2^, while, the lowest was obtained by the 100-CFA mix as 0.73 N/mm^2^.

#### 3.3.3 UPV test

**Fig. 11:**
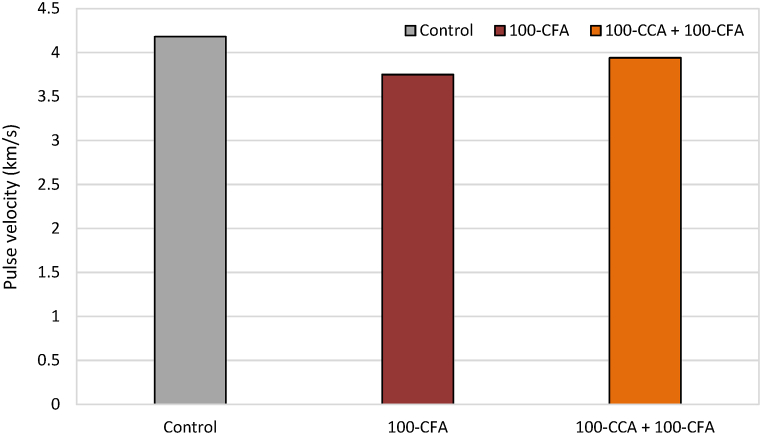
Pulse velocity results.

The results of the UPV test, which include the measured pulse velocities, were compiled and are shown in Fig. 11.

The UPV test conducted on the slab samples using the direct transmission method yielded an average pulse velocity of 3.95 km/s. This issue indicateed good uniformity and density of the tested slabs. Based on the results, NS1A, NS1B, and NS3A demonstrated that the quality of concrete was great for use. On the other hand, NS2A, NS2B, and NS3B revealed that the used concrete was good but may contain slight porosity.

#### 3.3.4 Schmidt hammer testing

**Fig. 12:**
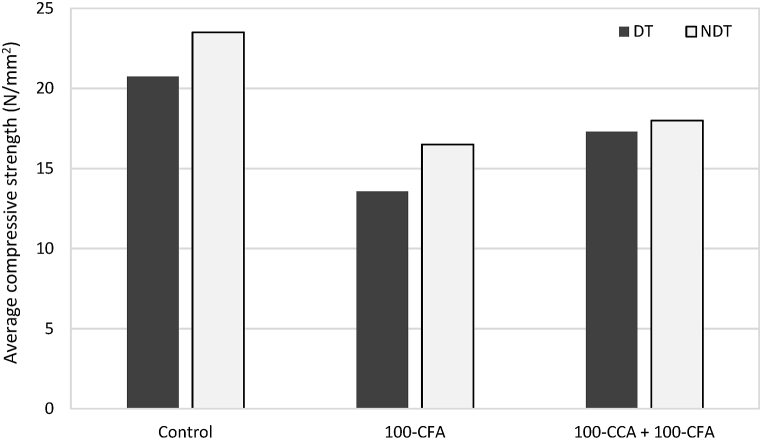
Comparison of average compressive strength values obtained from destructive test (DT) and non-destructive test (NDT) for various concrete mixes.

Fig. 12 presents the results of the Schmidt hammer testing performed on the slab samples. Each sample was subjected to three repeated impacts with the Schmidt hammer, and the rebound values were recorded. The average rebound value (R-value) and the corresponding estimated compressive strength were calculated using established conversion charts specific to the concrete mix design and testing standards.

The Schmidt hammer test conducted on the slab samples gave an average rebound value of 26.17, reflecting a moderate variation in the surface hardness across the samples. The results suggested NS1B had the best surface layer, while NS2B had the worst surface layer among the samples.

The variations in the rebound values of the samples can be attributed to several factors. First, differences in the concrete quality and consolidation during casting may lead to variations in the surface hardness. Additionally, variations in the curing conditions, including temperature and moisture, can impact the overall strength development of the slabs, thus, influencing the rebound values.

#### 3.3.5 Flexural strength test

Comprehending this aspect and exploring methods to enhance the flexural strength are essential for the optimal functioning of buildings and ensuring the safety of occupants. Structures such as bridges and floors are subjected to significant flexural loads due to heavy traffic and the loads they bear. These loads cause the slabs to bend or deflect under their weight. The amount of deflection or bending experienced by the slabs under flexural loading is influenced by various factors.

**Fig. 13:**
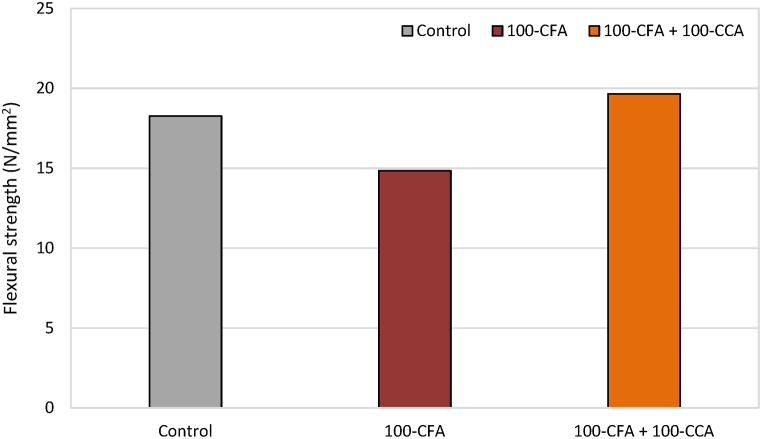
Flexural strength values of non-retrofitted slab samples.

**Fig. 14:**
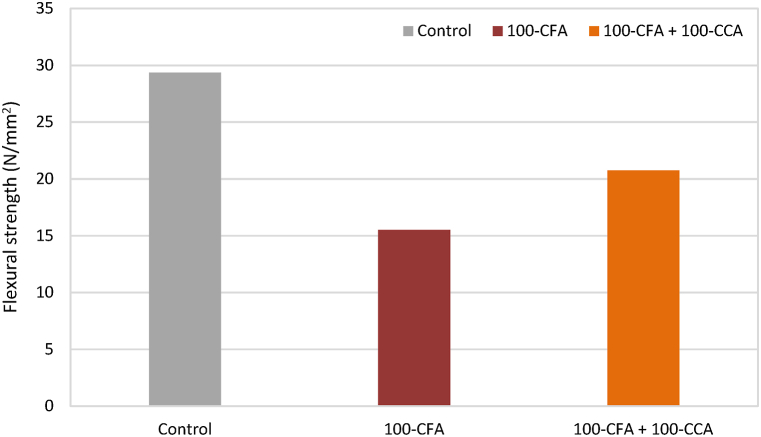
Flexural strength values of retrofitted slab samples.

It is a well-known fact that concrete exhibits limited strength when subjected to the tension and is comparatively stronger when subjected to the compression. However, the inherent deficiency of the flexural strength in concrete can be effectively improved through the incorporation of fibres. The presence of fibres introduces a crack arrest mechanism, enhancing the stiffness and toughness of concrete. This established phenomenon contributes to a noticeable increase in the flexural strength [25]. Now that challenges related to sustainability and global warming have come to light, bamboo is a popular topic of discussion. Fig. 13, Fig. 14 display the flexural strength values of the non-retrofitted slabs and retrofitted slabs, respectively.

The results of the flexural strength test revealed noteworthy differences between the non-retrofitted and retrofitted slab samples. Specifically, the flexural strength of the retrofitted samples exhibited a substantial increase compared to the non-retrofitted samples. The key findings are summarized below.

1. Conventional slab mix:
  i. Non-retrofitted slabs: Average flexural strength of 18.27 N/mm^2^.
  ii. Retrofitted slabs: Average flexural strength of 29.37 N/mm^2^.
  iii. This indicates a remarkable improvement of 60.76% in the flexural strength due to the retrofitting measures.
2. CFA mix:
  i. Non-retrofitted slabs: Average flexural strength of 14.85 N/mm^2^.
  ii. Retrofitted slabs: Average flexural strength of 15.51 N/mm^2^.
  iii. This demonstrates a modest improvement of 4.44% in the flexural strength owing to the retrofitting measures.
  iv. It is important to note that retrofitting of the slab did not significantly improve the CFA mix's very poor flexural strength.
3. 100-CCA + 100-CFA mix:
  i. Non-retrofitted slabs: Average flexural strength of 19.65 N/mm^2^.
  ii. Retrofitted slabs: Average flexural strength of 20.76 N/mm^2^.
  iii. This points out a slight improvement of 5.65% in the flexural strength due to the retrofitting measures.
  iv. The 100-CCA + 100-CFA mix was retrofitted, however, unlike the CFA mix, it did not considerably increase the mix's flexural strength.

These results showed how retrofitting actions could greatly increase the flexural strength of typical slab mixes. The little improvement for the CFA and 100-CCA + 100-CFA mixes, however, should serve as a reminder that the effect of retrofitting varies depending on the precise mix composition. Overall, the findings offer insightful information on how retrofitted and non-retrofitted slabs perform, assisting in the assessment and improvement of retrofitting procedures for greater structural integrity.

### 3.4 Crack patterns and modes of failures in samples

**Fig. 15:**
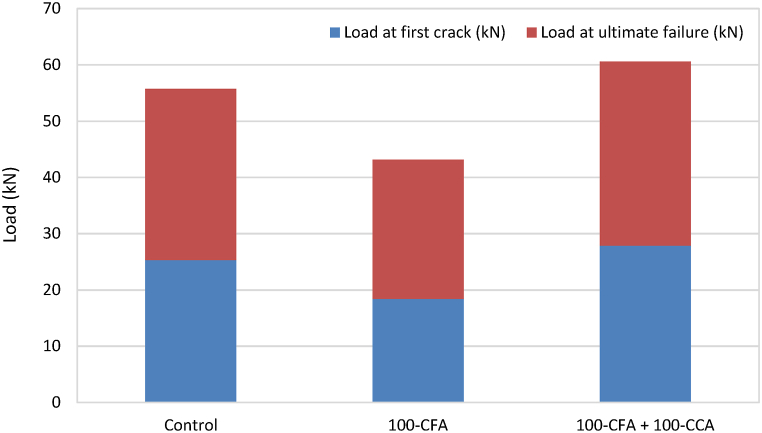
Load at first crack compared to ultimate failure load (non-retrofitted slabs).

**Fig. 16:**
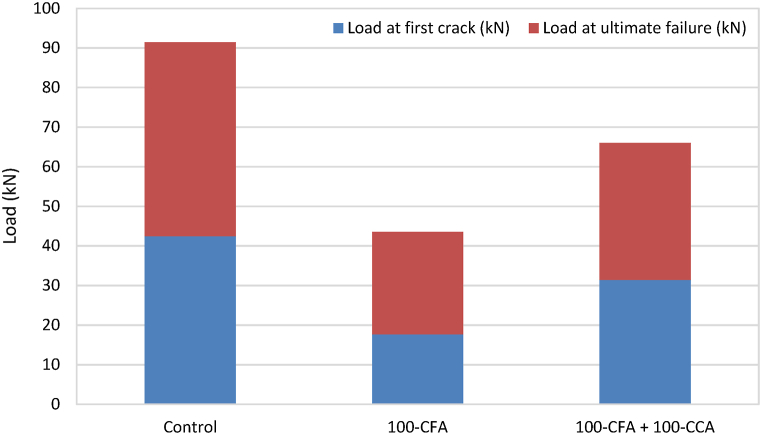
Load at first crack compared to ultimate failure load (retrofitted slabs).

Figs. 15 and 16 illustrate loads at first cracks compared to ultimate failure loads for the non-retrofitted slabs and retrofitted slabs, respectively. Table 3 provides the failure modes seen in the slab samples. The initial crack in the case of the control slabs, developed an average load of 42.45 kN, which was 67.79% higher than that of the slabs without retrofitting. For the retrofitted 100-CFA slabs, the first crack occurred at an average load of 17.65 kN, representing a decrease of 4.08% compared to the 100-CFA slabs without retrofitting. Notably, when subjected to load until the ultimate failure, there was only a marginal 4.44% improvement despite retrofitting with BFL. As for the retrofitted 100-CFA + 100-CCA slabs, the initial crack was observed at an average load of 31.4 kN, slightly higher by 12.75% compared to the mix without retrofitting.

**Table 3:** Failure modes observed in slab samples.

Designation of slabs	Observed failure modes
**NS1A**	Flexural failure (side and bottom)
**NS1B**	Flexural failure (side and bottom)
**NS2A**	Flexural failure (side and bottom)
**NS2B**	Flexural failure (side and bottom)
**NS3A**	Flexural failure (side and bottom)
**NS3B**	Flexural failure (side and bottom)
**RS1A**	Flexural failure (side) and bending of BFL
**RS1B**	Diagonal shear failure and bending of BFL
**RS2A**	Diagonal shear failure and bending of BFL
**RS2B**	Diagonal shear failure and bending of BFL
**RS3A**	Diagonal shear failure, bending of BFL, and debonding between BFL and slab
**RS3B**	Flexural failure (side) and bending of BFL

Among the non-retrofitted slabs, as the load was progressively applied until reaching the ultimate failure, flexural cracks in the form of flexural mode failure were observed across all the slab samples in this category. Additionally, at the bottom of all non-retrofitted slabs, a longitudinal flexural failure was seen, extending from one end of the slabs to the other.

In contrast, the retrofitted slabs exhibited three distinct types of failures: (i) diagonal shear failure, (ii) flexural failure, and (iii) debonding between BFL and the slabs. Among the retrofitted slabs, RS1B, RS2A, RS2B, and RS3A experienced failure primarily due to the diagonal shear, whereas RS1A and RS3B failed due to the flexural stress. Notably, RS3A displayed a significant occurrence of debonding between the slab and BFL. Despite reaching the ultimate failure, none of BFLs in the retrofitted slabs showed visible cracks or noticeable damage. They remained intact throughout the tests. However, it was observed that BFLs experienced bending at the support points during the tests.

**Fig. 17:**
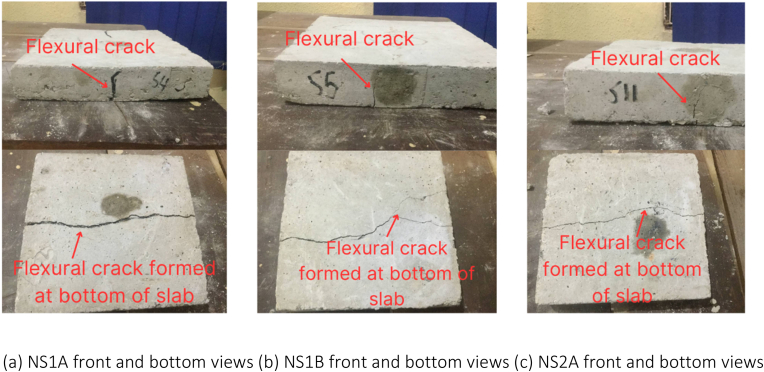
Failure modes and crack patterns for NS1A, NS1B, and NS2A.

**Fig. 18:**
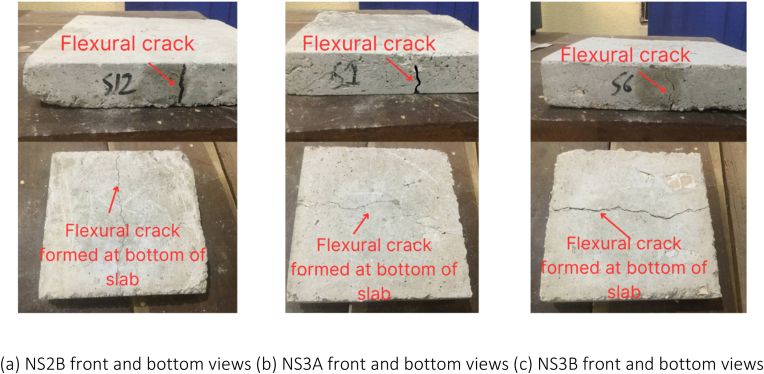
Failure modes and crack patterns for NS2B, NS3A, and NS3B.

**Fig. 19:**
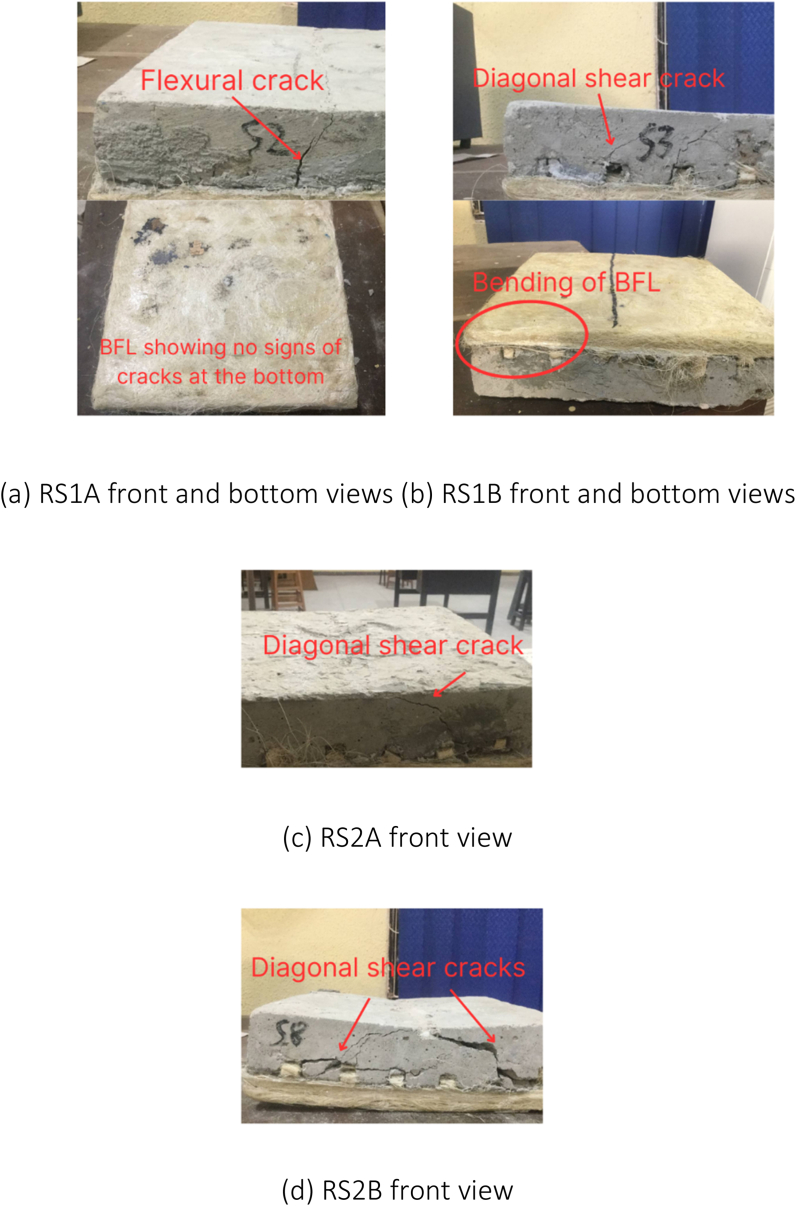
Failure modes and crack patterns for RS1A, RS1B, RS2A, and RS2B.

**Fig. 20:**
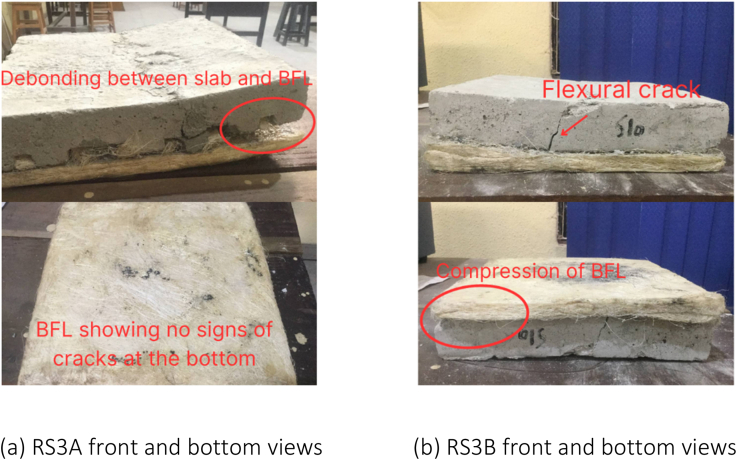
Failure modes and crack patterns for RS3A and RS3B.

When compared to the study conducted by Lim et al. [29] in which a metal decking composite slab was also utilized, the bamboo fibre laminated slab demonstrated a superior characteristic. In the event of cracks occurring, BFL beneath the slab can hold the broken concrete. This feature is highly significant in cases of building failures as it prevents the broken concrete from impacting residents, thereby providing residents with a longer window of time to safely evacuate the building during emergencies. The observed cracks in the slabs are depicted in Fig. 17, Fig. 18, Fig. 19, Fig. 20.

## 4 Conclusions

This study focused on the experimental investigation of the flexural performance of slabs that were retrofitted with BFL. The research used a variety of concrete mixtures, including normal concrete, 100-CFA mix, and 100-CCA + 100-CFA mix, to accord with sustainable structures. A number of tests were performed on these concrete mixes to see how structurally sound they were. Destructive testing such as splitting tensile and compressive strength tests were performed on concrete. Non-destructive testing was also done, including tests using the rebound hammer and UPV. Obtaining the flexural strength of the slabs utilized in this investigation was another aspect of the study. The slabs' crack patterns and modes of failures were carefully examined too.

The findings of the study can be summarized as follows:

• BFL greatly increased the flexural strength of the slabs. The control concrete mix exhibited a 60.76% increase in comparison to its non-retrofitted mix. The 100-CFA retrofitted mix had a slight improvement of 4.44%, and the 100-CFA + 100-CCA retrofitted mix had a 5.56% increase compared to the samples without retrofitting.
• The ultimate failure loads serve as confirmation of the overall strength improvement across the various mixes. Most significantly, the control mix had the ultimate failure load of 48.5 kN, which was a 59.3% increase in comparison to the slab sample without retrofitting.
• The non-retrofitted slabs demonstrated a number of failure modes, such as debonding between BFL and the slab, flexural failure, and diagonal shear failure.
• The retrofitted slabs indicated a variety of failure modes, such as the flexural failure, diagonal shear failure, and debonding between BFL and the slabs.
• There were no evident cracks or degradation in BFL. The only thing that bent was BFLs at the support locations.

Overall, the study revealed that retrofitting slabs with BFL greatly increases their flexural strength. The building sector can potentially use this knowledge to improve existing structures, especially those built using traditional concrete mixtures.

When evaluating the durability and long-term performance of the retrofitting material, it is important to note that BFL illustrated no obvious symptoms of damage or breaking. This information demonstrates that BFL has a good damage resistancewhich can be considered as a trustworthy retrofitting solution.

The study clarified that employing BFL reduced the performance of the 100-CFA mix by 4% in terms of the flexural strength. This finding expands our understanding by pointing out a specific situation in which using a BFL might not result in better performance. It encourages additional research on mixed compositions to enhance the retrofitting procedure.

## 5 Recommendations for future studies

The following suggestions have been made in light of the findings of the current research to improve and advance the findings as well as to offer fruitful directions for future study.

• Conduct additional research on the behaviour of various BFL types and combinations to assess how well they function when retrofitting slabs. Investigating various manufacturing processes as well as differences in laminate thickness and composition may be necessary.
• Carry out more research to assess the BFL's performance over the long term under various environmental circumstances, such as exposure to moisture, varying temperatures, and chemical agents.
• Determine whether applying BFL as a retrofitting option is economically feasible, taking into account aspects like material costs, installation procedures, and overall project expenses.
• Perform field research and real-world case studies to verify findings of the experimental research and determine whether BFL can be used to retrofit existing structures.
• Other highly sophisticated mechanical strength tests aside from the methods used in this research should be worked on for a more accurate analysis.
• Encourage further research and development in the field of sustainable retrofitting materials and techniques, aligning with the sustainable development goals to contribute to the advancement of environmentally friendly and resilient infrastructures.

## CRediT authorship contribution statement

**Paul O. Awoyera:** Writing – review & editing, Writing – original draft, Validation, Resources, Project administration, Methodology, Investigation, Conceptualization. **Fadi Althoey:** Writing – review & editing, Writing – original draft, Validation, Resources, Project administration, Methodology, Investigation, Conceptualization. **Alireza Bahrami:** Writing – review & editing, Writing – original draft, Validation, Resources, Project administration, Methodology, Investigation, Formal analysis, Conceptualization. **Pius U. Apuye:** Validation, Investigation. **L.M. Bendezu R:** Validation, Methodology. **Badr S. Alotaibi:** Validation, Project administration. **J.M. Prado M:** Validation, Formal analysis. **Mohammed A. Abuhussain:** Validation, Project administration.

## Declaration of competing interest

The authors declare that they have no known competing financial interests or personal relationships that could have appeared to influence the work reported in this article.
